# Technology Acceptance and Leadership 4.0: A Quali-Quantitative Study

**DOI:** 10.3390/ijerph182010845

**Published:** 2021-10-15

**Authors:** Monica Molino, Claudio G. Cortese, Chiara Ghislieri

**Affiliations:** Department of Psychology, University of Turin, Via Verdi 10, 10124 Turin, Italy; monica.molino@unito.it (M.M.); chiara.ghislieri@unito.it (C.G.)

**Keywords:** technology acceptance, work engagement, leadership 4.0, Industry 4.0

## Abstract

With the rapid advancement of Industry 4.0, new technologies are changing the nature of work and organizations. Nevertheless, technology acceptance is still an open issue and research, and practice interventions should investigate its antecedents and implement actions in order to reduce the risks of resistance and foster acceptance and effective usage of the new tools and systems. This quali-quantitative study was aimed at exploring perceptions about Industry 4.0 and its transformations and investigating job antecedents of technology acceptance. Whilst not many studies in the literature on technology acceptance have considered workers’ well-being, in this study, its association with work engagement has also been examined. The qualitative study used focus groups to collect perceptions of 14 key roles in a company that was implementing Industry 4.0. In the same company, the quantitative study involved 263 employees who filled in a questionnaire. The results confirmed that both job resources, namely supervisor support and role clarity, were antecedents of technology acceptance, which, in turn, was associated with work engagement. This study provides useful suggestions for interventions aimed at foster technology acceptance and workers’ well-being in companies that are facing Industry 4.0 transformations. Particularly, investments in both leadership 4.0 development and communication programs are essential.

## 1. Introduction

The rapid advancement of digital technologies is fundamentally changing the nature of work and organizations and has brought us into the Fourth Industrial Revolution [1] or Industry 4.0, term introduced for the first time in Germany in 2011 to describe a government-sponsored industrial initiative. The Fourth Industrial Revolution is represented by the development of new systems that combine physical, biological and digital concepts whose operations are integrated, monitored, and/or controlled by a computational core. They are called Cyber-Physical Systems (CPS) and are characterized by the blending of hardware and software that can interact with humans to complete tasks. Examples of technologies and systems that are leading the Industry 4.0 transformation are machine learning, big data, artificial intelligence, Internet of Things, cloud and mobile computing, biotechnology, nanotechnology, advanced robotics, exoskeletons, sensors and intelligent manufacturing, augmented reality, virtual reality, 3D printing [2]. For instance, robotics has changed the manufacturing industry in the last four decades; however, Industry 4.0 proposes a new set of collaboration defined by the human to machine interaction, that is the proximity between robots and human workers while sharing tasks [3]. Besides the manufacturing industry, which represents its origin, today, Industry 4.0 has become a more general concept with 99 Industry 4.0 neologisms found in the literature [4].

The synchronized development in different areas has created a perfect storm of new technologies that is rapidly and radically transforming almost all sectors [1], with the aim of improving, at the same time, the quality of both life and work for people and productivity for organizations. The use of new technologies leads to cost and time savings compared to higher quality of products and services; moreover, their use can improve health and safety in many environments [5]. According to Schwab [1], the Fourth Industrial Revolution differs from the previous ones for three reasons: (1) velocity, since it is evolving at an exponential rather than a linear pace; (2) scope, as it is disrupting almost all industrial sector; and (3) system impact, because it is transforming governance, management and production.

As with any revolution, in addition to the advantages, some disadvantages are also highlighted, mainly affecting workers and their employment prospects. In this regard, two schools of thought clash in the literature and in the general debate [5]. On the one hand, there are more drastic positions according to which the Fourth Industrial Revolution will lead to a decrease in jobs, with many occupations disappearing and being replaced by technologies [6]. According to the [7] OECD, 14% of occupations are at risk of full automation and 32% will undergo profound changes that will lead to skills obsolescence and make it increasingly difficult for people to find new jobs. This outlook, combined with the difficulty of trusting the unknown that automation and robots represent, may increase the risk of workers resistance [8]. These positions are contrasted with the more optimistic ones, according to which the current revolution, while on the one hand entailing an inevitable progressive exhaustion of some occupations, on the other hand, creates new employment opportunities by introducing occupations increasingly characterized by intellectual activities at the expense of physical effort and repetitiveness [9]. In the long term; therefore, it is expected that there will be more benefits than drawbacks [10], but for this to be the case, it is essential that significant investment is made immediately in training and social protection measures.

This study focuses on new technology acceptance in industrial sector, a topic that received great attention in the last years. However, its relationship with other individual factors has been under-considered while the impact of technology use on workers’ well-being has been suggested as an important direction for research [11]. Starting from these premises, this study contributes to the existing literature by investigating risks and opportunities of technology adoption with a particular focus on organizational antecedents of technology acceptance and its consequences for workers’ well-being. Among antecedents, the role of leadership deserves special attention, because it represents one of the most important areas of intervention to support the technological transformation. In the study, we used a quali-quantitative approach within a company adopting new technological systems. In the qualitative phase of the study, we used focus groups to explore and gather key roles’ opinions about Industry 4.0, risks and opportunities of technology adoption, leadership and skills of the future, and practical implications. In the quantitative phase of the study, we investigated some specific hypotheses about antecedents (supervisor support and role clarity) and consequences (work engagement) of technology acceptance.

### 1.1. Technology Acceptance

In recent years, understanding the dynamics that can influence people’s acceptance of new technologies has become increasingly important due to the widespread adoption of new devices and tools. The psychological and sociological literature on technology acceptance has collected several studies aimed at understanding what factors influence individuals’ intentions to use technology, and several models have been proposed [12]. The one that received the most attention is the Technology Acceptance Model (TAM) [13]. According to this model, which is derived from the Theory of Reasoned Action [14], individuals’ intention to use a new technology is predicted by their perception about that technology, and the actual use of the technology depends on the intention [13]. In particular, the intention to use a technology depends on two beliefs: “perceived usefulness, defined as the extent to which a person believes that using an IT will enhance his or her job performance and perceived ease of use, defined as the degree to which a person believes that using an IT will be free of effort” [15] (p. 275). In addition, the model accounts for the effect of external variables mediated by these two core beliefs.

Mathieson and colleagues [16] proposed a third belief for the TAM, namely perceived resources; they were defined as “the extent to which an individual believes that he or she has the personal and organizational resources needed to use an information system” (p. 89). They are an important component in explaining technology acceptance because they highlight the role of the environment in which the technology is implemented. The perception of receiving organizational support can have a positive influence on acceptance and intention to use a new technology; on the other hand, organizational barriers can hinder acceptance.

This resources component was also taken up by the Unified Theory of Acceptance and Use of Technology (UTAUT) [17], which proposes four constructs to explain the acceptance of new technologies: performance expectancy (the belief that the technology will help with job performance), effort expectancy (the perception of ease in using the technology), social influence (the perception that significant others are convinced that the technology should be used), and facilitating conditions (the perception of organizational support and resources).

More recently, Kaasinen and colleagues [18] proposed the Worker-Centric Design and Evaluation Framework for Operator 4.0, which differs from previous models as it also considers workers’ well-being in the design and evaluation of new technological systems and tools. Since human-centricity is a relevant aspect for Industry 4.0 and the factories of the future [18], the investigation of technology acceptance and use should also consider individual outcomes; for this reason, we referred to this model in our study. The Worker-Centric Design and Evaluation Framework for Operator 4.0 drew on one of the most popular work-related well-being models, proposed by Danna and Griffin [19]. According to this model, well-being in the workplace is influenced by antecedents such as personality traits, occupational stress and work setting; in turn, well-being at work impacts on individual and organizational outcomes. The Kaasinen and colleagues framework [18] integrates the role of technology and proposes that the individual’s experiences with a new technological tool (acceptance and usability) has antecedents at the individual, organizational, and environmental levels. Antecedents include the original context of use in terms of work environment, the role, task and goal involved by the use of the tool, organizational resources or barriers, and the worker characteristics. Moreover, worker’s experience with the tool can affect his/her work-related well-being (e.g., job satisfaction, work engagement, and job motivation) and organizational benefits (e.g., optimized processes, quality, productivity, and company image) [18].

This model is consistent with both the job demands-resources (JD-R) theory [20] and the effort-recovery theory [21]. According to the JD-R theory, each work environment has its own characteristics that can be grouped into two dimensions: “job demands are those physical, psychological, social or organizational aspects of the job that require sustained physical and/or psychological (cognitive and emotional) effort or skill”; “job resources refer to those physical, psychological, social, or organizational aspects of the job that are either/or: functional in achieving work goals; reduce job demands and the associated physiological and psychological costs; stimulate personal growth, learning, and development” [22] (p. 312). Whereas job demands are the most important predictors of exhaustion, job resources predict work engagement, which is defined as a positive work-related state of mind, characterized by vigor, dedication and absorption [23]. The effort-recovery theory [21] assumes that workers’ motivation is enhanced by resources provided by the environment: if the work environment offers several resources, it is more likely that the worker dedicates his/her efforts to the task and successfully achieves work goals.

The Worker-Centric Design and Evaluation Framework for Operator 4.0 [18] draws on the relationship between antecedents (including job demands and resources) and outcomes (including work engagement) and proposes a mediation of the experience with technology. In the case of the introduction of a new technology, if the work environment is rich in resources, such as support from the supervisor, clarity of task and role, communication and feedback, or training, the worker will have a better chance of understanding why and how to use the new tool and accepting it. In turn, having a positive experience with the tool and recognizing its usefulness and advantages might further improve the motivational process and other individual and organizational outcomes.

### 1.2. Leadership 4.0

Leadership 4.0 is one of the neologisms introduced following the advent of Industry 4.0 [4,24] and refers to the need of specific and advanced skills for those leaders who are involved in the frontline management of the pros and cons of the Fourth Industrial Revolution and in the definition of the strategies for both the functioning of the organizations and the involvement of workers [11].

Kelly [25] has described the leadership evolutions in relation to the different industrial revolutions which have taken place over time. According to his analysis, at the end of the 18th century, the first industrial revolution was accompanied by a 1.0 leadership, defined as charismatic, which could be embodied only by one person with specific personality and quality traits, which were innate and exceptional. At the beginning of the 19th century, the second industrial revolution, modeled by the scientific organization of work, introduced a 2.0 directive leadership, which shifted from innate traits to typical behavioral models of the top-down approach. In the 70s, with the third industrial revolution, we find a 3.0 relational leadership, which moves from transactional to transformational [26], which orients collaborators towards objectives, simultaneously promoting autonomy, creativity, and collaboration. Finally, the fourth industrial revolution, which is taking place in these years, asks for a leadership that possesses already known features—firstly, relational skills—but also new competencies to efficiently act in the digital and automated world with a particular focus on education, innovation, and the management of change. Therefore, leadership 4.0 can support the transition towards the Industry 4.0, reconcile chaos and unpredictability with the stability of organizational processes, and allow for fundamental discoveries and innovations to keep up with technological progress and global competition [27]. According to Kelly’s definition [25], leadership 4.0 needs to be a reactive type of leadership, which encourages the experimentation of new technologies, embraces exchanges and reacts to the feedback of the collaborators, and guarantees opportunities and resources for continuous learning [28]. From this perspective—and in line with the followership theories [29]—critically constructive dialogue between leaders and followers based on the change itself and on innovation is essential in order to support the proactivity of all people involved.

Leadership 4.0 must combine the ability to manage the implementation of new technologies, with the ability to involve people; in this phase, there is a need for leaders who, in addition to the implementation of new technologies, can also guarantee their acceptance and their correct use on behalf of their employees [30]. Oberer and Erkollar [31] proposed the 4.0 leadership matrix to identify different possible leadership styles based on one’s interests in technologies and innovation, on the one hand, and interest in people on the other. At the crossroads between a vocation towards technology and innovation and attention to people, we find digital leadership—the most effective and productive type of leadership—which tries to link new organizational models and people’s needs in an appropriate way. Excessive focus on technologies at the cost of people describes a technological leadership, which focuses predominantly on the ability to establish how new technologies can be used to guarantee higher added value; excessive focus on people describes a social leadership, which is careful and pays attention particularly to the well-being of employees. Lastly, a freshmen leadership is not particularly oriented towards innovation or people. Instead, it maintains the focus on traditional production structures and the finalization of the product.

According to Oberer and Erkollar [31], the 4.0 digital leader should have the ability (1) to shift from a fixed cycle performance evaluation system towards a more flexible and focused system, providing constant feedback; (2) to assign tasks based on competencies and needs; (3) to stimulate and manage change at all levels; (4) to manage the processes, to assess activities and people (based on their potential and competencies) beyond the confines of a single product; (5) to create an open and collaborative climate in which mistakes are opportunities for growth and conflicts can be managed; (6) to create a transparent environment in which information circulates effectively, responsibility is shared and people are proactive; and (7) to understand that innovation can be learned and obsolete structures can be transformed through the involvement of multidisciplinary teams, using creative processes. The 4.0 digital leadership, therefore, is by nature a fast and reactive inter-hierarchical and inter-functional approach, oriented towards people and teams; it is collaborative and highly geared towards innovation [31]. The aspect of communication is also essential. First, it starts with the willingness to inform all staff on the reasons for change, on how it will occur and what its impact will be on the entire organization [32]. Subsequently, opening a communication channel with each individual employee is essential to ensure clarity on how new tools modify their work and their role, and give space for employees to ask questions and address any perplexities. On the one hand, this approach increases the chances that employees will develop a positive attitude towards new technologies; on the other hand, it contributes to making them feel involved [32].

Summing up, the main responsibilities of leaders, at this historic moment, are: (1) the creation of a digital culture in which people positively embrace transformation; (2) the definition of the conditions required to guarantee the requalification and the development of the employees’ necessary digital competences; (3) the effort to attract and to keep workers with specialized technical profiles; (4) sharing winning strategies for technological development [33,34]. The focus on technologies is undeniable and widely recognized; however, in terms of relationships with people, there is still a need to adopt leadership styles that historically have proven effective as they are based on relationships and non-pedagogical support, which are not aimed at creating dependence within the relationship but are oriented towards the development of autonomy and empowerment. Therefore, in the era of Industry 4.0 and in the leader–follower dynamic, the elements of the leader–member exchange [35] or transformational leadership [26] models are confirmed and required, with the aim of making people grow within an educational relationship, allowing them to express their leadership potential.

### 1.3. Study Aims

Following the above-mentioned theories, models and considerations, the aim of this study was to identify job resources that are capable of promoting technology acceptance and consequently work engagement. The study followed a quali-quantitative approach. First, focus groups were conducted in a specific organization, which was implementing some technological transformations, in order to collect and analyze the opinions of key roles about Industry 4.0, risks and opportunities of technology adoption, leadership, and skills of the future. Then, employees were involved in a quantitative study and the data was used to test the following hypotheses.

Following The Worker-Centric Design and Evaluation Framework for Operator 4.0 [18], we investigated the role of two specific job resources as determinants of new technology acceptance. As described previously, the role of leaders is critical in promoting good worker response to technologies [31]; in this study, we considered a specific component of leadership, namely supervisor support, as a job resource. To the best of our knowledge, the relationship between social support and technology acceptance have received little attention by researchers so far. Carreiro and Oliveira [36] found that transformational leadership factors, such as vision and personal recognition, were positively related to the firm’s intention to adopt mobile cloud computing. Lee and colleagues [37], in a study about health technology use, found that participants with higher social support were more likely to accept technology use. Masood and Lohdi [38], in their study about the adoption of a statistical software by students, came to the conclusion that teacher support was of greater importance than specific knowledge of software.

In addition, we included role clarity as a job resource in the model, which is defined as the degree to which workers feel it is clear what is expected from their roles and behaviors [39]. The introduction of new technologies alters workers’ roles, the tasks they perform and the way they perform them. The chance to deal positively with a change depends on the understanding of the change itself and of the expected effects that the change will have for individuals and their work [40]. Previous studies have shown that workers who felt informed and involved in decisions related to technology change reacted more positively to the changes than colleagues less involved [41]. Moreover, if workers perceive that the technology will help them to perform and will be helpful and easy to use, the likelihood of accepting and adopting it increases [42].

**Hypothesis 1 (H1).**  *supervisor support and role clarity are positively related to technology acceptance.*

According to the JD-R theory [20], we also examined the direct relationship between the two job resources and work engagement. Both supervisor support and role clarity are among the most investigated job resources by studies that applied the JD-R theory to explain the motivational process and antecedents of work engagement [43].

**Hypothesis 2 (H2).**  *supervisor support and role clarity are positively related to work engagement.*

**Figure 1:**
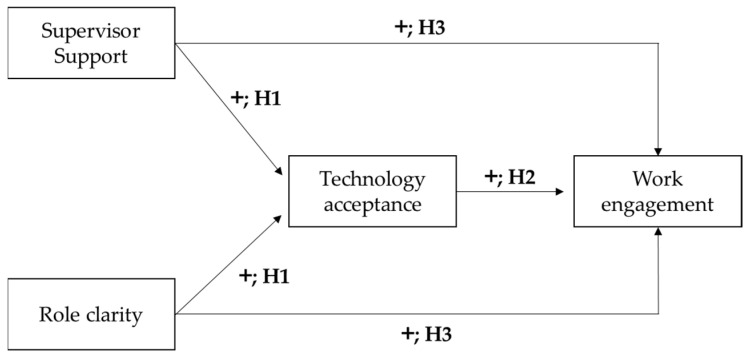
The hypothesized model.

Finally, we investigated the relationship between technology acceptance and workers’ work engagement and the mediation of technology acceptance between the two job resources and the final outcome. These hypotheses are based on the previously described Worker-Centric Design and Evaluation Framework for Operator 4.0 [18]. If the work environment provides resources such as supervisor support and role clarity, we expect that the workers will be more willing to use the new technology, and in turn, accepting to use it and recognizing it as a further resource for the performance of work activities will increase work engagement. A previous study found a mediational role of technology acceptance between resilience, a personal characteristic, and opportunities for information and training on the one hand, and work engagement on the other hand [32]. In general, as far as we know, these dynamics have received little attention in the literature, since studies neglected the consequences of technology acceptance on individual outcomes. Figure 1 shows the study hypotheses.

**Hypothesis 3 (H3).**  *technology acceptance is positively related to work engagement.*

**Hypothesis 4 (H4).**  *technology acceptance partially mediates the relationship between job resources (supervisor support and role clarity) and work engagement.*

## 2. Materials and Methods

### 2.1. Procedure and Participants

The quali-quantitative study has been realized in an Italian manufacturing company involved in Industry 4.0 innovations (especially exoskeletons and innovative robots within the plants). The project, designed in collaboration with the Health, Safety and Environment Department, received approval from the Company’s Board of Directors and the trade unions. Moreover, it has been carried out in line with the Helsinki Declaration [44] and the Italian data protection regulation.

For the qualitative study, 14 participants have been involved using focus groups. They included plant key roles (Human Resources Manager, Plant Manager, Health and Safety Manager) and employees from the training department. Almost all of them were men (12 = men; 2 = women).

In the quantitative study, informed consent was obtained from all participants. They have been informed about the study’s aims, anonymity, data protection, and the voluntary participation to the study. Questionnaires were administered on-site during working hours in the presence of a researcher and have been returned in drop-boxes. The participants represented the 42% of the whole population; it was a representative sample per gender, age and organizational departments.

The sample included 263 employees; 83% were male and 17% were female. The mean age was 41.44 (SD = 12.01). Among them, 53% had a high school diploma, 31% had a middle school diploma, and 15% a Bachelor’s or Master’s degree; the remainder had a lower educational level. Participants were blue-collar (66%) and white-collar (34%). The mean seniority on the job was 20.97 years (SD = 13.05).

### 2.2. Measures for the Quantitative Study

Work engagement was measured through 8 items [45]; an example item is “I find my work to be a positive challenge” (Likert scale from 1 = Totally disagree to 5 = Totally agree). The Italian version of the scale (already used in previous studies, e.g., [32]) presented good psychometric properties: the confirmatory factor analysis confirmed the one-factor structure [χ^2^ (18) = 53.19, *p* < 0.001, CFI = 0.95, TLI = 0.93, RMSEA = 0.08, SRMR = 0.04] with factor loadings ranging between 0.42 and 0.79. Cronbach’s alpha was 0.83.

Technology acceptance was measured using 5 items taken from the Subjective Acceptance Questionnaire [46]; an example item is “The use of technology and automation systems increases my professional effectiveness” (Likert scale from 1 = Totally disagree to 5 = Totally agree). In this case also, the Italian version of the scale (already used in previous studies, e.g., [32]) presented good psychometric properties: the confirmatory factor analysis confirmed the one-factor structure [χ^2^ (4) = 14.55, *p* = 0.06, CFI = 0.98, TLI = 0.94, RMSEA = 0.07, SRMR = 0.04] with factor loadings ranging between 0.49 and 0.92. Cronbach’s alpha was 0.74.

Supervisor support was detected with 4 items taken from the questionnaire proposed by INAIL (the Italian National Institute for Insurance against Accidents at Work) to assess and manage the risks related to work-related stress [47]; an example item is “I receive information from my supervisor that helps me in the work” (Likert scale from 1 = Never to 5 = Always). Cronbach’s alpha was 0.90.

Role clarity was measured through 5 items (INAIL questionnaire) [47]; an example item is “My tasks and responsibilities are clear to me” (Likert scale from 1 = Never to 5 = Always). Cronbach alpha was 0.80.

### 2.3. Data Analysis

To analyze qualitative data, the method of the Grounded Theory has been used [48,49]. Data gathered through interviews and focus groups was conceptually labelled and the concepts were then categorized and organized by identified dimensions. The conceptual labels have been translated into 7 categories, described in the Results section.

As for quantitative data, descriptive analysis, Pearson correlations, and Cronbach’s alpha coefficients were performed using the statistics software SPSS 26 (IBM, Armonk, NY, USA). The structural equation model (SEM) and the confirmatory factor analysis were performed using Mplus 7 (Muthén & Muthén, Los Angeles, CA, USA). The estimation method was Maximum Likelihood (ML); in the model, we controlled for participants’ age according to previous studies that found more resistance to new technology among older workers (e.g., [50]). The following goodness-of-fit criteria have been suggested in the literature to assess a model [51]: the χ^2^ goodness-of-fit statistic; the Root Mean Square Error of Approximation (RMSEA); the Comparative Fit Index (CFI) and the Tucker Lewis Index (TLI); the Standardized Root Mean Square Residual (SRMR). Finally, the significance of the indirect effects was tested using the bootstrapping procedure [52].

## 3. Results

### 3.1. Qualitative Results

The conceptual labels collected through the focus group have been translated into seven categories. In the following subsections, each category is described.

#### 3.1.1. Industry 4.0: Opportunities and Risks

This category gathers the definitions provided by the participants on the general theme of the Industry 4.0 and their opinions regarding its advantages and opportunities, on the one hand, and its disadvantages and risks on the other. From the answers provided, it is clear that knowledge on the topic is limited within the company and has not been explored by the participants themselves.

“I imagine that behind the definition of Industry 4.0 lies much more and I am unaware of what this may be, to be completely honest”.

[Plant Key Role]

Based on the definitions provided, the general thought appears to be that Industry 4.0 is the concept of collaboration between men and technology which places the role of the individual in a privileged position. The Industry 4.0 is considered as a fundamental opportunity to allow different worlds to integrate with the aim of providing maximum efficiency and satisfying financial, ethical, and social needs. Regarding advantages and opportunities, what emerges clearly is the perception of technology at the service of the worker; new technologies will be used to lighten the operator’s workload and to make his/her tasks easier. More specifically, these tools are viewed as devices which can reduce physical fatigue, especially for older workers. Therefore, technology is considered to be a resource that is also meant to provide additional support for ergonomics.

On the other hand, some of the disadvantages and risks have been described. These are important to understand potential resistance on behalf of workers to the introduction of new tools. First, there are fears pertaining to the impact that new technologies could have in the working context in terms of the substitution and reduction of staff. Furthermore, considering specifically the generational gap, there is a fear that the 4.0 Industry will lead to effects of social exclusion, as not all people can easily adapt to technological changes; consequently, this could lead to a gap, particularly between young people who are used to using technology at work and in their personal lives, and more senior workers.

“If in the past, only three people worked in the workplace and now there’ll only be two people with a collaborative robot, then it’s logical to think that this will have some kind of impact”.

[Plant Key Role]

“It is something that needs to be accessible to everybody, because there is also a strong risk that people will fall behind, unable to keep up with the new technologies, and become more and more excluded”.

[Plant Key Role]

#### 3.1.2. How Will Workers React?

We asked participants to describe how, in their opinion, workers will react to the implementation of new devices. What emerges is that most workers have not been informed of what 4.0 Industry actually is; only some of them have heard people talk about it and others have expressed faint curiosity towards it. The common and shared opinion is that there is some confusion as to what the changes would entail and what their consequences would be.

“In my experience, we’ve talked about it in training activities. People don’t know much. They are very curious but also skeptical, but they really don’t know much”.

[Trainer]

According to what participants in the focus groups have reported, the staff are both hopeful and fearful with regard to these new changes. Some people perceive the danger that new devices entail, and they feel that all roles will be automated, which will increase control and job loss; others view these changes as functional in helping them to lighten their workload and physical fatigue. So, these devices are seen as both allies and enemies.

“We often talked to workers about robotization and the introduction of automation in the industry. Of course, there’s always some sadness or regret in saying that people’s jobs could be taken away. However, everyone recognizes the fact that, if we look at it from a different perspective, robots could also take on tasks that are too stressful, cumbersome, or dangerous for us, and could work in dangerous environments. This is a unanimous thought”.

[Trainer]

“There’s the fear of losing work or thinking that the machine could control them somehow. And this is something they would never want”.

[Trainer]

#### 3.1.3. Differences Linked to Personal Backgrounds

In the focus groups, we also investigated the potential presence of differences linked to personal backgrounds in the opinions given on the 4.0 Industry, for example, gender, age, seniority and type of job or task performed. The results show that the most important differences concern age and seniority, as more senior workers have a different conception of work in general as compared to younger workers. More specifically, older workers have more experience, have more awareness and have already acquired the habit of performing their tasks in a certain way; young people, instead, tend to be more flexible, more open to change, to adapting, and are more willing to learn. Regarding gender differences, it is not possible to make a comparison due to the limited number of women working in the company.

“We could say that the older generations are a little more skeptical. Young people tend to be more open; they experience it, almost as if it were an evolution in their day to day lives, but they also know very little about the changes”.

[Trainer]

#### 3.1.4. Acceptance or Refusal of the New Devices

Taking into consideration the real innovations that the company was introducing, participants were asked specific opinions about the use of collaborative robots and exoskeletons. Regarding collaborative robots, there were diverging opinions. What was deemed fundamentally important were the concepts of trust and trustworthiness. Only if robots are perceived by workers as trustworthy can there be positive reactions. If the contrary occurs, then they will be perceived as devices towards which people should nurture negative feelings, and, consequently, their introduction will lead to hostile reactions. Furthermore, and specifically in reference to these kinds of devices, there is the fear of losing one’s job and being replaced. There is also the fear that the robot could become a model worker who never tires or sets a work pace that workers will have to adapt to. In order to avoid resistance, introducing the new robots through an effective and transparent communication process is advisable, with particular emphasis on the advantages, not only for the company but also and especially for the workers, guaranteeing the respect of the features and limits of the people.

“People need to develop complete trust towards these tools and create a relationship of trustworthiness with the robot. This is the big challenge, I think, in the use of collaborative robots of this kind”.

[Plant Key Role]

“In terms of acceptance, I can’t envisage specific problems, even though the risk I foresee could be that the robot will be seen as man’s substitute. This is an issue”.

[Plant Key Role]

In terms of exoskeletons, there are varying levels of acceptance. In some cases, the positive outcomes that these devices could bring are highlighted, particularly in terms of ergonomics and reducing fatigue. Consequently, we assume that there could be positive reactions. However, negative factors are also reported, and these could present an obstacle when introducing exoskeletons. These include the invasion of personal space. The management of one’s personal space is a rather delicate issue, as exoskeletons could be considered an intrusion or invasion not only the worker’s workstation—as happens with collaborative robots—but also his or her physical sphere, as an exoskeleton needs to be worn on the worker’s body. The concept of control also carries some critical and delicate reflections. The main concerns that workers have regard how much they can control the device and how much the device may control them and limit their freedom. Finally, there is also mention of the possible discriminatory effect that using these devices could generate, especially when these devices are used on people with physical disabilities, who could be targeted and, therefore, discriminated against or marginalized.

“Many technicians have talked about these exoskeletons, and they say it’s like a bicycle with assisted pedaling. They’re not afraid, and I do not think they would react with fear”.

[Trainer]

“Planning needs to be connected also to how much I can control this mechanism, or how much it controls me, the awareness and the certainty of being able to control it, to curb my fear, the fear that it might harm me and the fear that I may not be able to control it, or that he is controlling me”.

[Trainer]

“With regard to the exoskeleton, the only thing that needs to be addressed is the physical management of the tool. The ability to use it and wear it. There could be people who feel uncomfortable having something on their body”.

[Plant Key Role]

#### 3.1.5. The Skills of the Future

This category collects the opinions of participants on competencies that they deem necessary to interact with these devices and adapt to this new reality. With regard to hard skills, there seem to be contrasting opinions. Some people say that there are no specific competencies required; on the contrary, assisted modalities that are offered by technological devices will reduce people’s thinking and initiative, as they will only be asked to perform the tasks that they are given and to repeat the operations.

“The 5.0 Industry may only need baboons. What need is there for humans? The more we move forward, the less is asked of people”.

[Trainer]

“To avoid errors to ensure quality, we need someone who does not think. Someone who takes the piece—the only piece that is there—puts it in its position where the light is turned on”.

[Trainer]

Others, on the other hand, believe there is a need to acquire new competencies or to fill the gaps that currently exist. Reference is made to the acquisition of technical competencies because the new workspace is almost completely automated. Consequently, there is a need to maneuver devices appropriately, respond to the technological commands or repair damage to the system. Currently, technology can be considered a further tool available to people, and as such, people need to be able to use it as best as possible.

“I’m not all that convinced that people using these devices should not have more training than they have now. We’ve gone from people using a hammer, cutter or a screwdriver to people managing complex machinery. We need to change the professional profile”.

[Trainer]

“Certainly, there will be more need for more technical vision of the role of the worker who in the future will have a technical/technological role”.

[Plant Key Role]

Regarding soft skills, opinions are aligned: the most important competences that these new changes require are flexibility and proactivity. These two competences are essential to adapt in the best way, and in the shortest time possible, to the changes that are taking place in an era that is characterized by insecurity and uncertainty.

#### 3.1.6. Leadership 4.0

Regarding managers, we expect to see the development of new technical competencies on their behalf, particularly knowledge of the work cycle of the machinery that they will be interfacing with and the activities that will be taking place.

“Alongside the changes that the workers will have to make, it is also essential that the managers are aware of the types of technologies that are going to be used because the best way to manage people is to know what they have to do and in what conditions”.

[Plant Key Role]

In addition to this, the leaders must display a guidance and proactive attitude; they should try to bring everyone on to the same level through the acquisition and transfer of a common language to use internally within their group. They must give people a sense of change and transformation. They must know how to integrate and promote the different competences, which are seen as a strength for the success of the community, and consequently, the entire organization. Furthermore, they should be capable of managing the present and communicating the vision of the future, being those who carry people forward, and do not leave anybody behind.

#### 3.1.7. Interventions to Communicate the Change and Support Acceptance

The need to communicate what is happening was unanimously viewed as crucial to reduce negative reactions or refusal and promote openness with the aim of ultimately gaining acceptance. Therefore, a communicative style which is clear and transparent is preferred, and the ultimate objective of this communicative style is to create a common language on all organizational levels to reduce and potentially eliminate possible misunderstandings and ambiguities. To this end, the advantages and the consequences of the Industry 4.0 are to be made clear, and workers are to be given the opportunity to express their doubts and curiosities, and to gain answers. In this way, workers will feel considered and well regarded within their working context, and their active participation and involvement will be facilitated through concrete proposals such as a media campaign using screens in the areas where staff members meet and gather. On those screens, there will be videos that have been tailormade to teach people about the changes. Alternatively, a poster system around the company to show through images and concepts that are easy to understand the features and advantages of the new technologies. Furthermore, in order to ensure that exceedingly high expectations are managed or unrealistic fantasies are curbed, the creation of a laboratory or a showroom in which workers can see and experiment with the prototypes of the new devices has also been suggested.

“Awareness is an important key. I explain to you what this is, and you get to talk about it and give me feedback, tell me your opinion. So, any potential fears that you have will be addressed. At that point, there is no slamming on the brakes and there is no putting up a wall. On the contrary, there is active participation on behalf of the workers”.

[Trainer]

#### 3.1.8. Training

In terms of training activities, we suggest a practical training component, in addition to theoretical type training. The main focus of training should be the acquisition of technical-specialistic competencies and the transferability of the teachings on a manual level. The most effective method seems to be training on the job, which allows employees to acquire the technical and manual skills they need to use and maneuver the different devices introduced, through training and the initial shadowing of a more expert colleague. Shadowing an expert is particularly useful because, in the early stages, it will be useful in allowing the operator to observe the modality and method through which the devices are used; subsequently, the expert can provide support as a guide and a supervisor of the different activities performed and be an advisor in times of difficulty.

Then there is the problem of how training is an investment for the company, both in terms of cost and man hours. To address this criticality, the suggestion is to initially provide training only to managers; this way, management will be prepared to answer to any type of questions that the workers bring their way, and it will also have a top-down effect.

“We train the managers first so they know what is coming, and they can then be ready to address any question. We go from the manager and work down the ladder until reaching the newest employees”.

[Plant Key Role]

### 3.2. Quantitative Results

This section describes the quantitative results related to the test of the hypotheses. Table 1 shows means, standard deviations, and correlations between the variables. Work engagement showed a positive correlation with technology acceptance, supervisor support, role clarity and age. Technology acceptance showed a positive correlation with supervisor support and role clarity and a negative one with age.

**Figure 2:**
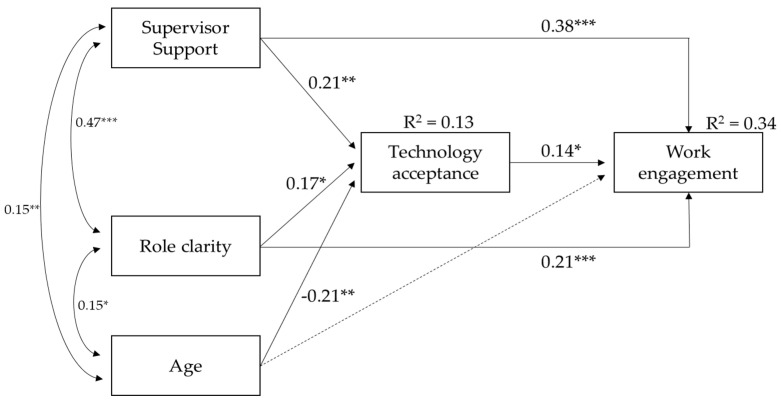
The final model (standardized path coefficients). Discontinuous lines indicate non-significant relationships. *** *p* < 0.001; ** *p* < 0.01; * *p* < 0.05.

The estimated model, which is shown in Figure 2, fits to the data well: χ^2^ (0) = 0, *p* < 0.001, CFI = 1.00, TLI = 1.00, RMSEA = 0.00, SRMR = 0.00. In the model, both the supervisor support and role clarity showed a positive relationship with technology acceptance. In turn, technology acceptance was positively related to work engagement. Moreover, both supervisor support and role clarity had a direct positive association with work engagement. Age showed a negative association with technology acceptance. The model explained about 13% and 34% of the variance in, respectively, technology acceptance and work engagement. Table 2 showed the results of the bootstrapping procedure used to test indirect effects. They were significant in all cases confirming the partial mediation of technology acceptance.

## 4. Discussion

Recent technological innovation is expected to improve the productivity, efficiency and quality of work in several industrial sectors [1]; however, not accepting new technology can have consequences for both organizations and employees’ well-being [53]. According to this perspective, the present study intended to investigate organizational antecedents and individual consequences of technology acceptance, using a quali-quantitative approach. It contributes to the general knowledge of Industry 4.0 in the following areas: (1) it is one of the first studies that investigate the consequences of technology acceptance on a specific dimension of workers well-being, namely work engagement; (2) it empirically confirmed the crucial role of leaders in managing and fostering the Industry 4.0 transformations; (3) to do so, it applied a quali-quantitative approach which can be considered particularly valuable in this area of study. Indeed, the qualitative part of the study allowed a deep investigation of the topics of interest collecting workers’ opinions and bringing about specific suggestions for practice and new questions for future research. In addition, the quantitative part of the study permitted the application of a model, the Worker-Centric Design and Evaluation Framework for Operator 4.0 [18], which, unlike other models widely used in the literature, supports the investigation of technology use taking into account also worker-related outcomes.

The first hypothesis (H1) postulated that both supervisor support and role clarity would be positively related to technology acceptance, and it was confirmed by the quantitative data. Receiving professional and personal support from one’s supervisor is fundamental to providing what Oberer and Erkollar [31] refer to as digital leadership: in addition to paying attention to the implementation of new technologies and systems, leaders should also be mindful of the needs of their followers. According to the worker-centric approach, if the worker is able to interact with his/her supervisor, ask questions and feel supported, he/she will be more motivated to accept and use new technological tools to perform work activities. This result is in line with those findings found in other sectors, such as health and education, where social and supervisor support was positively related with technology acceptance [37,38]. The importance to invest in leadership as a resource helpful for the Industry 4.0 transformation has been highlighted also from qualitative results. In the focus groups, participants have suggested how important it is that leaders develop technological and digital skills. Moreover, they emphasized that leaders are responsible for the involvement of all workers, that they should be supportive and attentive to their needs concerning the change and their work.

At the same time, it should be clear for employees how the change will affect their work activities and roles, and they should be given clear instructions about expectations. Role clarity is especially important in contexts where it is not possible to increase autonomy and decision-making latitude in the workplace, but it may be possible to ensure role clarity [54], as is the case with the introduction of new technologies: workers cannot decide whether and how to use the new system or tool, but they can receive clear instructions about how their role will change and how it will be affected by the introduction of the technology. Understanding the change and its effects on work roles and activities is a necessary condition for accepting the change itself [40]. In this term, participants of the focus groups highlighted the importance of activating information and communication processes aimed at making employees involved and clear about the change and its implications [32]. In this way, the organization can make the facilitating conditions [17] to welcome workers’ doubts and fears and provide proper answers. These suggestions are in line with previous studies that found higher levels of technology acceptance for workers who felt informed and involved in the change compared with colleagues less involved [41,42].

The second hypothesis (H2), which was confirmed, considered the direct relationship between the two job resources and work engagement. Our results were consistent with the motivational process of the job demands-resources theory that has widely proved the direct effect between job resources, such as supervisor support and role clarity, and work engagement [20]. In the literature, work engagement is considered as one of the most important outcomes of organizational well-being as it showed positive associations with many other outcomes such as job performance, in-role and extra-role performance, service quality, and customer loyalty [55].

According to hypothesis 3 (H3), which was also confirmed, technology acceptance showed a positive association with work engagement. The relationship between technology acceptance and well-being has received little attention so far [32], although the concept of human-centricity is dominant to the Industry 4.0 argument. Our findings confirmed that a positive attitude towards a technology not only promotes its effective and positive use, but also plays a role in motivational dynamics that make employees more positive, willing to invest efforts, and persistent in the face of challenges or problems [23].

Finally, hypothesis 4 (H4) was also confirmed, as we found a partial mediation of technology acceptance between the two job resources and work engagement. In a context of significant change and transformation, such as the one considered in our study, and more generally in all companies facing the challenges of Industry 4.0, work engagement also depends on how individuals perceive the new technologies introduced and to what extent they accept them. Thus, job resources can have a positive effect on work engagement through their ability to promote technology acceptance. All these findings open up important and concrete lines of intervention that will be discussed below, taking into account the information gathered through the focus groups.

### Limitations and Directions for Future Research

The study has some limitations. The first one is that the quantitative study was cross-sectional; thus, causal inferences about relationships between variables are not allowed. Nevertheless, the qualitative data supported the understanding of the quantitative results and confirmed the hypothesized directions. Future longitudinal studies are needed to improve the understanding of technology acceptance dynamics in industries, prioritizing research that monitors over the time and evaluates the adoption of specific tools in specific working contexts. Another limitation is that the quantitative study only included self-reported data, which increases the risk of common method-bias [56]. Other reported data (e.g., from peers and supervisors) and/or objective data should be considered in this type of study.

In addition, a multilevel approach to the study of leadership in organizations is highly praised, in order to observe the investigated effects on a team level basis. Nevertheless, the collection of multilevel data in such a context is not always possible and efforts to strengthen the collaboration between research and industry would be also necessary. Other limitations concern the measures applied in the quantitative study for the investigation of study hypotheses. Particularly, it would be necessary to develop, validate and apply a specific measure able to detect leadership 4.0, considering the two core assets suggested by Oberer and Erkollar [31], namely technology and people. The investigation of technology acceptance should also refer to specific tools and roles, while the employees involved in this quantitative study performed different work activities and used different technological tools. A further idea for future research is related to the need to clarify the differences between Industry 4.0 and Industry 5.0 and their implications, with the latter considered an evolution of the former which emphasizes the synergy between humans and autonomous machines [57]. Although Industry 4.0 is still in its growing phase, according to some scholars, many technology and industry leaders are looking ahead to the Fifth Industrial Revolution, and the role of Work and Organizational Psychology will be even more important to contribute to this field by investigating psychological dynamics and effects on workers motivation and well-being.

Finally, the small sample size of the focus groups should be mentioned as a limitation. Almost all key organizational roles were included; however, future qualitative studies should expand to other roles and, above all, should involve workers directly involved in the use of the new technologies. Nevertheless, as a point of strength, it should be acknowledged that the quantitative study considered a large representative sample of workers employed in the same company.

## 5. Conclusions

The field of the relationship between Industry 4.0 and workers’ well-being is still in its nascent stages; this study, using a quali-quantitative approach contributes to the literature, providing useful suggestions for research and practice. The rapid advancement of new technologies in organizations is inevitable, and organizations need to embrace this transformation. In this process, employees play a crucial role, and fostering positive attitudes toward new technologies is essential to guarantee acceptance and, in turn, high motivation and work engagement. Our results highlighted that supervisor support and role clarity are important factors to support this process.

From a practical point of view, adequate opportunities for information should be offered to all workers when technological changes are implemented. As suggested by focus-group participants, communication and information programs can be used to anticipate the change, deal with doubts and fears, and promote collective awareness. Communication channels such as videos, posters and slogans should be disseminated in the workplace, using images and short, simple and direct messages to communicate the characteristics of the new tools, their benefits for workers and the implications they will have on work activities. The fear of losing the job should also be addressed through this communication process.

Training on the job to develop the needed technological skills, to learn how to use the new tools and to understand how they impact workers’ role is essential. Moreover, all workers could be involved in workshops and classroom training about Industry 4.0 and technological transformations, to make them more aware and opened to change. To overcome the generational gap, senior workers could receive specific training on digital skills, referred to both professional and personal contexts. Finally, specific training should be offered to all people in leadership positions, to develop their ability to focus on both technological improvements and workers’ needs while they make strategies for the future and manage the technological change. To offer them a specific and targeted support, also mentoring and coaching are appropriate interventions.

## Figures and Tables

**Table 1:** Means, standard deviations, Cronbach’s alphas and correlations among study variables.

	1	2	3	4	5
1. Work engagement	*−*				
2. Technology acceptance	0.26 **	*−*			
3. Supervisor support	0.53 **	0.26 **	*−*		
4. Role clarity	0.43 **	0.22 **	0.47 **	*−*	
5. Age	0.12 *	−0.19 **	0.15 *	0.14 **	*−*
M	3.49	3.84	3.69	4.08	41.44
SD	0.77	0.79	1.05	0.69	12.01

Note. Cronbach’s *α* on the diagonal. ** *p* < 0.01; * *p* < 0.05.

**Table 2:** Indirect effects using bootstrapping (2000 replications).

Indirect Effects	Est.	S.E.	*p*	CI 95%
Sup. Sup.→Tech.→WE	0.06	0.02	0.042	(0.02, 0.07)
Rle clarity→Tech.→WE	0.04	0.01	0.048	(0.01, 0.06)
Age→Tech.→WE	−0.03	0.01	0.037	(−0.06, −0.01)

Note. All parameter estimates are presented as standardized coefficients. CI = confidence interval. Sup. Sup.: supervisor support. Tech.: technology acceptance. WE: work engagement.

## Data Availability

Data supporting reported results are available upon request to the first author.
